# Attitudes and beliefs about musculoskeletal pain and its association with pain neuroscience knowledge among physiotherapy students in Israel

**DOI:** 10.1186/s13584-018-0266-4

**Published:** 2018-12-15

**Authors:** Shmuel Springer, Hadas Gleicher, Hila Hababou

**Affiliations:** Department of Physical Therapy, Faculty of Health Sciences Ariel University, 40700 Ariel, Israel

**Keywords:** Pain, physiotherapy students, Attitudes, Education

## Abstract

**Background:**

Physiotherapy has a vital role in helping patients manage and overcome musculoskeletal pain. Healthcare providers’ beliefs about pain are associated with the beliefs of their patients. This study evaluated the attitudes, beliefs and level of pain neuroscience knowledge among Israeli Bachelor-level physiotherapy students.

**Methods:**

First-year (*n* = 29, before pain course), second-year (*n* = 28, immediately after pain course and before clinical placements), and fourth-year (n = 28, post-clinical placements) physiotherapy students completed the Health Care Providers’ Pain and Impairment Relationship Scale (HC-PAIRS, range 15–105, lower scores indicate a more positive attitude) to assess pain attitudes and beliefs. The Neurophysiology of Pain Questionnaire (NPQ, range 0–19, higher scores indicate more pain-related knowledge) was also completed to measure pain neuroscience knowledge. Two separate one-way ANOVAs with post hoc analyses were used to compare HC-PAIRS and NPQ results between the three groups of students. Pearson correlations were determined between HC-PAIRS and NPQ.

**Results:**

HC-PAIRS scores of the first-year students were significantly higher than those of second- and fourth-year students (*p* = 0.011, *p* < 0.001, respectively), with no difference between second- and fourth-year students; indicating that first-year students had less-positive attitudes toward the ability of individuals with musculoskeletal pain to function. Similarly, NPQ scores showed that first-year students differed from second- and fourth-year students (*p* < 0.001, p < 0.001, respectively). The HC-PAIRS and NPQ correlation among the fourth-year students yielded a moderately negative association (*r* = − 0.462, *p* = 0.01), indicating that pain neuroscience knowledge was associated with less belief that chronic pain justifies disability.

**Conclusions:**

A specific curriculum about pain during physiotherapy undergraduate education contributes to a more positive evidenced-based attitude to musculoskeletal pain and patient function. The association between pain neuroscience knowledge and positive attitudes and beliefs regarding pain were enhanced after clinical placements, demonstrating that learning improves when integrated into practice. Due to the impact of pain training and the expected benefits to patient care, health policy decision makers and educators should verify that the pain curriculum is current with the best research evidence. Future studies with larger samples that include students from other healthcare disciplines, including medicine are warranted.

## Background

Caring for patients with chronic pain is a major challenge in healthcare. Estimates of the prevalence of chronic pain range from 10% to over 40%, including in Israel [1, 2]. Traditionally, understanding the pathophysiology of chronic musculoskeletal pain and its treatment was based on the biomedical model. However, it the past few decades, recognition of the role of psychosocial factors in chronic pain mechanisms and their importance in managing pain has increased substantially [3]. Furthermore, a biopsychosocial approach was found to be superior to a biomedically-focused approach for treating many chronic musculoskeletal pain conditions [4, 5].

Using a biopsychosocial approach, physiotherapy plays a vital role in helping patients manage and overcome musculoskeletal pain [4]. Negative beliefs regarding pain are associated with increased disability for the individual with chronic pain [6]. Thus, reconceptualizing beliefs and knowledge about pain through education is a key element in this method of treatment [7]. Helping patients change their perceptions may decrease fear avoidance and catastrophizing, reduce disability, and improve physical performance [8].

The ability to modify a patient’s concepts about pain is strongly determined by the clinician’s perspective about illness and pain. There is ample evidence that healthcare providers’ beliefs about pain can affect the beliefs of their patient [6, 9]. Moreover, clinicians’ perceptions influence their adherence to clinical practice guidelines recommended for people with chronic pain. Healthcare professionals with a biomedical orientation or high fear avoidance beliefs are more likely to advise patients to limit work and physical activities and are less likely to follow current treatment guidelines [9]. Consequently, it is crucial for clinicians to have adequate understanding of the neurophysiology of pain and the role of psycho-social factors in order to implement the biopsychosocial approach.

Physiotherapists’ concepts of pain are established during their formal undergraduate education. Several previous studies have evaluated physiotherapy students’ attitudes toward pain [10, 11]. Latimer et al. [10] showed that following a 16-h teaching module on chronic low back pain (LBP), Australian physiotherapy students were less likely to agree that chronic LBP justifies impairments and disability. Similarly, Ryan et al. [12] reported that fourth-year UK physiotherapy students had more positive attitudes towards functioning in individuals with back pain than did first-year physiotherapy students. Ferreira et al. [11] demonstrated that compared with Australian physiotherapy students, Brazilian physiotherapy students agreed more strongly with the notion that pain justifies limitation of activities in chronic LBP patients.

The International Association for the Study of Pain (IASP) recommends all entry level physiotherapy programs to include a specific pain curriculum [13]. However, none of the previous studies evaluated the effect of a designated pain course on attitudes of physiotherapy students toward pain. It also important to test the association between practitioners’ pain neuroscience knowledge to perception and thoughts regarding chronic pain. Furthermore, clinical placements are an integral component of part of student education. They encounter practical, hands-on experience and have the opportunity to deepen their knowledge. Thus, students’ attitudes and beliefs should be evaluated with respect to clinical placements.

Therefore, the aim of the present study was to describe attitudes and beliefs regarding musculoskeletal pain among first-year (before pain course), second-year (immediately after pain course and before clinical placements), and fourth-year (two years after pain course and after clinical placements) physiotherapy students in Israel. A second aim was to investigate whether the level of pain neuroscience knowledge is associated with attitudes and perceptions regarding musculoskeletal pain.

## Methods

### Procedure

The study was approved by the Ethics Committee of the Faculty of Health Sciences, Ariel University, Israel. A cross-sectional survey study design was used.

The Health Care Providers’ Pain and Impairment Relationship Scale (HC-PAIRS) **(**Appendix**)** [14] was used to assess students’ attitudes and beliefs about musculoskeletal pain. The HC-PAIRS scale contains 15 statements suggesting that the impairments and disability found in patients with chronic LBP are directly attributable to pain. The respondent indicates on a Likert scale how much they agree with each statement, represented by 1 = completely disagree to 7 = completely agree. Possible scores range from 15 to 105; the higher the score, the greater belief that pain justifies disability and activity limitations. The instrument was widely used by studies that measured the attitudes and beliefs of health sciences students [10–12]. A systematic review of tools measuring attitudes and beliefs, indicated that the HC-PAIRS demonstrated adequate internal consistency (Cronbach’s alpha 0.78 to 0.84), test-retest reliability and was consistent with other relevant measures [15].

The Neurophysiology of Pain Questionnaire (NPQ) **(**Appendix**)** [7] was used to evaluate understanding of pain neuroscience. The NPQ tests pain-related knowledge, focusing on the biological mechanisms underpinning pain [7]. It contains 19 items that were originally based on postgraduate pain medicine exam papers. The response options are true, false, or undecided, each correct response receives one point, whereas incorrect or undecided responses score zero points. Higher scores indicate greater understanding of pain neurophysiology. Various studies have used the NPQ to assess pain-related knowledge [7, 16, 17]. The NPQ has acceptable internal consistency (Person Separation Index = 0.84), which suggests that it is sensitive enough to distinguish between high and low performers [18].

The NPQ and HC-PAIRS questionnaires were translated into Hebrew in accordance with the Word Health Organization process of translation and adaptation of instruments [19]. The survey was conducted during the second semester of 2017. It included first-, second- and fourth-year physiotherapy students at Ariel University. The Bachelor-level physiotherapy study program at Ariel University is spread over four years (including clinical placements). A 26-h pain course is given during the second semester of the second year. The aim of the course is to enable entry-level physiotherapists to understand the neurophysiology of pain in normal and pathological conditions, as well as the psychosocial and environmental components of pain and their impact on the pain experience across the life span. Upon completion of the course, students should be able to develop an evidence-based management program in collaboration with their future patients. All students in each class (i.e., first-, second- and fourth-year) were asked to complete both questionnaires, either in person or by email. The participants provided written informed consent before completing the questionnaires.

### Data analysis

Reliability of the translated HC-PAIRS and NPQ questionnaires was determined by computing Cronbach’s alpha. Descriptive statistics were used to present questionnaire results (mean ± standard deviation). Normal distribution was tested using Shapiro-Wilk test. Then, two separate one-way ANOVAs were used to compare HC-PAIRS and NPQ results between the three groups of students (first, second, and fourth year). Post hoc analysis with Bonferroni corrections was used to examine pairwise differences, as appropriate. To test the association between pain neuroscience knowledge and attitudes and beliefs regarding pain, Pearson correlations were determined between HC-PAIRS and NPQ for the entire sample of students, as well as for each year, separately. Correlations were interpreted as suggested by Cohen [20] where 0.10 represents a weak or small association; 0.30 a moderate correlation; and 0.50 or greater a strong or large correlation. SPSS (SPSS Inc., Chicago, Illinois) was used for statistical analyses. Significance level was set at *p <* 0.05.

## Results

Among a study population of 139 students, 85 (61%) returned completed questionnaires. Of these, 29 were first year students, 28 s year, and 28 fourth year.

Cronbach’s alpha was 0.657 for the HC-PAIRS and 0.731 for the NPQ. The HC-PAIRS and NPQ scores are presented in Table 1.

**Table 1:** HC-PAIRS and NPQ scores (mean ± SD) and correlations in each student group and in the entire sample

Students	HC-PAIRS	NPQ	HC-PAIRS - NPQ correlation
First-year (n = 29)	65.45 ± 5.82	7.48 ± 2.92	0.400 *p* = 0.031
Second-year (*n* = 28	58.61 ± 9.69	13.39 ± 1.93	−0.043 NS
Fourth-year (n = 28)	56.25 ± 9.98	12.82 ± 2.57	−0.462 *p* = 0.01
Entire sample (*n* = 85)	60.16 ± 9.44	11.19 ± 3.66	−0.342 *p* = 0.001

The one-way ANOVA on the NPQ and HC-PAIRS measure between the groups yielded a significant group effect (*p* < 0.001 for both). Post hoc test on the HC-PAIRS score indicated significant differences between first-year and second-year students (*p* = 0.011) and fourth-year students (*p* < 0.001), with no difference between second- and fourth-year students. This indicates that first-year students had less-positive attitudes regarding the ability of individuals with musculoskeletal pain to function. Similarly, post hoc test on the NPQ score showed significant differences only between the first-year students to second- and fourth-year students (*p* < 0.001, p < 0.001, respectively), implying that first-year students had lower level of pain neuroscience knowledge. The results of the post hoc comparisons are presented in Table 2.

**Table 2:** Post hoc comparisons

		Second-year	Fourth-year
HC-PAIRS	First-year	*p* = 0.011	*p* < 0.001
	Second-year		NS
NPQ	First-year	*p* < 0.001	*p* < 0.001
	Second-year	–	NS

Pearson correlations between the HC-PAIRS and NPQ scores (Table 1) yielded moderate negative associations for the entire sample (*r* = − 0.342, *p* = 0.001) as well among the fourth-year students (*r* = − 0.462, *p* = 0.01) indicating that greater understanding of pain neuroscience was associated with reduced belief that chronic LBP justifies disability. In contrast, in the first-year student group, a positive correlation was found (*r* = 0.400, *p* = 0.031), indicating that their pain neuroscience knowledge was associated with a belief that chronic LBP justifies disability.

## Discussion

The findings of the current study indicate that after being taught a specific pain curriculum, the attitudes and beliefs regarding chronic pain among Israeli physiotherapy students changed significantly. Following the pain course, students agreed less with the concept that chronic LBP justifies disability and activity limitations. This change in attitude was preserved two years later, after completing clinical placements. These findings are consistent with the study of Latimer et al. [10] who showed that Australian physiotherapy students had lower HC-PAIRS scores after exposure to a specific teaching module. The HC-PAIRS questionnaire refers to chronic back pain. While Latimer’s et al. teaching module was specific for back pain, the results from the current study demonstrate that a designated general pain course (i.e., not specific to spinal pain education) can positively alter students’ attitudes regarding a specific type of chronic pain (i.e., LBP). This may imply that after a pain course, students will likely support a biopsychosocial approach that promotes patients developing an active strategy to cope with various musculoskeletal pain conditions. Ryan et al. [12] showed that fourth-year physiotherapy students had more positive attitudes towards ability of individuals with back pain to function than first year physiotherapy students did (HC-PAIRS scores: 57.4 vs. 66.6). That study did include any specific module about pain. The authors concluded that physiotherapy education based on the biopsychosocial model leads to positive student attitudes towards functioning in individuals with chronic pain, more than any single module about pain does. Further studies should evaluate wheatear a specific pain course should be taught during physiotherapy education to increase positive attitudes towards functioning in individuals with chronic pain.

Like the HC-PAIRS score, the results obtained indicate that pain neuroscience knowledge (i.e. NPQ) improved immediately after the pain course and remained at the same level after clinical placements. However, moderate association between pain neuroscience knowledge to attitudes and beliefs regarding pain was found after the clinical placements (fourth-year students), but not immediately after the pain course. Learning does not occur by acquiring theoretical knowledge only. During clinical practice, students enter the “real-world” encountering social and cultural conditions that differ from those found in a classroom. The reinforced correlation between pain knowledge and belief after clinical practice is in accordance with previous studies demonstrating that learning is enhanced when integrated into practice [21, 22]. Yet, none of the previous studies evaluated the association between practitioners’ pain neuroscience knowledge to their perceptions and thoughts regarding chronic pain.

It should be noted that among the first-year students, better pain knowledge was associated with a belief that chronic pain justifies disability. A possible explanation may be related to the significantly lower level of knowledge in the first year (NPQ = 7.48) compared to the second and forth years (NPQ = 13.39 and NPQ = 12.82, respectively). Furthermore, the findings indicate that prior pain neuroscience understanding alone cannot ensure positive, proactive attitudes toward managing patients with chronic pain. Thus, as suggested by the IASP, comprehensive pain education for students should reconceptualize unhelpful beliefs about pain, such as fear avoidance [13].

To the best of our knowledge, this is the first report about pain beliefs and knowledge among Israeli students. The HC-PAIRS score of the first-year students (65.45) was similar to the scores of first-year physiotherapy students in the UK (66.6) [12]. Yet, third- and fourth-year Brazilian physiotherapy students had similar HC-PAIRS scores (66.4) [11] and Australian third-year physiotherapy students (who did not receive a module about pain) had much better scores (53.3) [10]. Pain beliefs are strongly influenced by culture [23]. Previous studies have shown that students from different countries and cultural backgrounds differ in their perceptions of chronic pain [11, 23]. Therefore, these differences may be related to cultural issues and to the way chronic pain is perceived, as well as to different approaches during physiotherapy graduate education.

Several studies indicated differences in the level of pain neurophysiology knowledge, as well as in pain beliefs and attitudes among various healthcare professionals and medical students [16, 23–25]. For example, Ali and Thomson [24] reported that final year physiotherapy students had greater knowledge of chronic pain than final year medical students did. Briggs et al. [25] reported that physiotherapy students’ beliefs about the consequences of LBP and the relationship between LBP and impairment were in closer alignment with evidence, as compared to medicine, occupational therapy, and pharmacy students. Additionally, surveys revealed that the content of pain education for undergraduate healthcare professionals is varied and in most cases woefully inadequate [26, 27]. Insufficient training might result in low confidence and low perceived competency to address pain. Physicians reported not receiving effective training regarding the role of biopsychosocial factors and thus, felt low self-efficacy in addressing and managing biopsychosocial issues [28]. Chronic musculoskeletal pain poses a substantial challenge to the medical community. Greater numbers of older people and lifestyle changes throughout the world with increasing obesity and reduced physical activity indicate that the burden of pain will increase dramatically in the next few decades [29].

In Israel five faculties have an entry-level physiotherapy program, Tel Aviv University, Ben Gurion University, University of Haifa, Ariel University, and Zefat Academic College. All programs include a 26-h designated pain curricula, except Ben Gurion University which will initiate a program by the 2019 academic year. Nevertheless, the pain curricula are varied and not all address affective and cognitive dimensions of pain. It should also be noted that Israeli physiotherapy students have less pain education when compared with other countries. In the UK physiotherapists have 38 h of pain education [26], in Canada 41 h [30], and in the United States 31 h [31]. Health policy decision-makers and educational facilities such as universities should adopt a broad strategic plan to provide healthcare professionals with the skills they need to manage pain effectively and sustainably. Specifically, a need for broader and standardized pain curriculum in entry-level physiotherapy programs that will enable students to develop clinical competencies based on up-to-date concepts of pain is needed. As suggested by the IASP, the curriculum should be designed with appropriate emphasis on the current theories and science of pain that consider psychological and social factors of pain and pain management. It may also be advisable to develop advanced programs (e.g., Master’s degree) intended for healthcare professionals who want to specialize in the field of pain management.

A byproduct of this study are Hebrew versions of the NPQ and HC-PAIRS questionnaires. Cronbach’s alpha values obtained from the Hebrew versions were 0.731 for the NPQ and 0.657 for the HC-PAIRS. A recent methodological review describing how Cronbach’s alpha is used within published studies indicated that there is no clear consensus on the most appropriate labels for reporting and interpreting this value [32]. The review presented a range of terms and values used by authors to interpret calculated alpha values. Among these values and terms were, satisfactory (0.58–0.97), acceptable (0.45–0.98), and sufficient (0.45–0.96). Although the Cronbach’s alpha value obtained from the Hebrew HC-PAIRS was slightly lower than the original version (Cronbach’s alpha 0.78 to 0.84, 15], it seems that the internal consistency values obtained in the current study for both questionnaires are acceptable. Nevertheless, further studies that will evaluate the psychometric properties of these translated versions are warranted.

There are number of potential limitations to the current study. It included a relatively small sample of physiotherapy students from one university, that might not be representative of all physiotherapy students in Israel. Future studies with larger samples that include students from other healthcare disciplines and medical students should be conducted to evaluate attitudes and beliefs regarding pain. As with all surveys, there might be potential selection bias, as those with extreme pain beliefs or strong pain knowledge might have been more likely to participate. However, this bias seems unlikely, as the participants’ responses were varied and presented different approaches. Furthermore, although the response rate of 61% is considered acceptable [33], future research should seek to gain a higher response rate. Finally, information regarding age, gender, and previous or current chronic pain was not collected. Yet, it should be noted that similar studies that tested pain attitudes and beliefs among physiotherapy students did not report that these factors influence results [10, 11].

## Conclusions

A designated pain course during physiotherapy undergraduate training can modify students’ attitudes towards functioning and coping strategies among individuals with chronic pain. An association between pain neuroscience knowledge and positive evidence-based attitudes and beliefs regarding pain was found after clinical placements, but not immediately after the pain course. This indicates that learning is enhanced when integrated into practice. Due to the impact of pain training and the expected benefit to patient care, health policy decision-makers and educators should verify that the pain curriculum is up to date with the best research evidence available.

## Appendix

**Table 3:** Health Care Providers’ Pain and Impairment Relationship Scale (HC-PAIRS)

		Totally Agree	Largely Agree	Agree Somewhat	Neutral	Disagree Somewhat	Largely Disagree	Totally Disagree
1	Chronic back pain patients can still be expected to fulfill work and family responsibilities despite pain							
2	An increase in pain is an indicator that chronic back pain patients should stop what they are doing until the pain decreases							
3	Chronic back pain patients cannot go about normal life activities when they are in pain							
4	If their pain would go away, chronic back pain patients would be every bit as active as they used to be							
5	Chronic back pain patients should have the same benefits as the handicapped because of their chronic pain problem							
6	Chronic back pain patients owe it to themselves and those around them to perform their usual activities when their pain is bad							
7	Most people expect too much of chronic back pain patients, given their pain							
8	Chronic back pain patients have to be careful not to do anything that might make their pain worse							
9	As long as they are in pain, chronic back pain patients will never be able to live as well as they did before							
10	When their pain gets worse, chronic back pain patients find it very hard to concentrate on anything else							
11	Chronic back pain patients have to accept that they are disabled because of their chronic pain							
12	There is no way that chronic back pain patients can return to doing the things that they used to do unless they find a cure for their pain							
13	Chronic back pain patients find themselves frequently thinking about their pain and what it has done to their life							
14	Even though their pain is always there, chronic back pain patients often do not notice it at all when they are keeping themselves busy							
15	All of chronic back pain patients’ problems would be solved if their pain would go away							

**Table 4:** Neurophysiology of Pain Questionnaire (NPQ)

		True	False	Undecided
1	Receptors on nerves work by opening ion channels (gates) in the wall of the nerve			
2	When part of your body is injured, special pain receptors convey the pain message to your brain.			
3	Pain only occurs when you are injured.			
4	The timing and intensity of pain matches the timing and number of signals in nociceptors (dangerreceptors)			
5	Nerves have to connect a body part to your brain in order for that body part to be in pain			
6	In chronic pain, the central nervous system becomes more sensitive to nociception (dangermessages)			
7	The body tells the brain when it is in pain			
8	The brain sends messages down your spinal cord that can increase the nociception (danger message) going up your spinal cord.			
9	The brain decides when you will experience pain			
10	Nerves adapt by increasing their resting level of excitement.			
11	Chronic pain means that an injury hasn’t healed properly.			
12	Nerves can adapt by making more ion channels (gates)			
13	Worse injuries always result in worse pain			
14	Nerves adapt by making ion channels (gates) stay open longer			
15	Second-order nociceptor (messenger nerve) post-synaptic membrane potential is dependent on descending modulation			
16	When you are injured, the environment that you are in will not have an effect on the amount of pain you experience.			
17	It is possible to have pain and not know about it.			
18	When you are injured, chemicals in your tissue can make nerves more sensitive			
19	In chronic pain, chemicals associated with stress can directly activate nociception pathways (dangermessenger nerves).			

## Abbreviations

HC-PAIRS: The Health Care Providers’ Pain and Impairment Relationship Scale
IASP: The International Association for the Study of Pain
LBP: Low back pain
NPQ: The Neurophysiology of Pain Questionnaire
